# Enhancing trehalose production via *Bacillus* species G1 cyclodextrin glucanotransferase mutants: modifying disproportionation characteristics and thermal stability

**DOI:** 10.3389/fmicb.2024.1500232

**Published:** 2024-11-19

**Authors:** Bobo Miao, Di Huang, Tengfei Wang, Hongling Liu, Zhifeng Hao, Haibo Yuan, Yi Jiang

**Affiliations:** ^1^State Key Laboratory of Biobased Material and Green Papermaking (LBMP), Qilu University of Technology (Shandong Academy of Sciences), Jinan, Shandong, China; ^2^Key Laboratory of Shandong Microbial Engineering, College of Bioengineering, QiLu University of Technology (Shandong Academy of Sciences), Jinan, Shandong, China; ^3^Yantai Zhaoyi Biotechnology Co., Ltd, Yantai, China

**Keywords:** directed evolution, maltodextrin, maltose, protein engineering, transglycosylation

## Abstract

Inefficient conversion of small molecule maltooligosaccharides into trehalose greatly affects the cost of the production of trehalose by double enzyme method [maltooligosyl trehalose synthase (MTSase) and maltooligosyl trehalose trehalohyrolase (MTHase)]. This study used directed evolution to increase oligosaccharide utilization by the cyclomaltodextrin glucanotransferase (CGTase) from *Bacillus* species G1. This enzyme was chosen for its adaptability and stability in trehalose production. Model analysis revealed that the hydrogen bond distance between the N33K mutant and maltose reduced from 2.6 Å to 2.3 Å, increasing maltose affinity and boosting transglycosylation activity by 2.1-fold compared to the wild type. Further mutations improved thermal stability and optimum temperature, resulting in the N33K/S211G mutant. Consistent results from repeated experiments showed that the N33K/S211G mutant increased trehalose yield by 32.6% using maltodextrin. The results enhanced the double-enzyme method formed by MTSase and MTHase for trehalose production. Overall, we have identified optimal catalytic conditions, demonstrating significant potential for industrial-scale trehalose production with enhanced efficiency and cost-effectiveness.

## 1 Introduction

Trehalose is a bioactive protective agent that widely used in the food, pharmaceutical (Chen et al., 2022; Wang et al., 2024) and cosmetic industries (Li et al., 2017). In the biotechnology filed, trehalose enhances efficiency of microbial wastewater treatment (Ma et al., 2023), improves industrial fermentation processes, and aids in extracting valuable chemicals from biomass. Current methods for enzymatic trehalose synthesis primarily rely on a dual-enzyme system involving maltooligosyl trehalose synthase (MTSase) and maltooligosyl trehalose trehalohyrolase (MTHase), effectively converting maltodextrin into trehalose (Wang et al., 2023). This method is favored for its cost-effectiveness and simplicity, yielding substantial trehalose alongside minimal glucose and maltose (Chen et al., 2020; Huang et al., 2024). However, maltooligosaccharides (MOS) with low degrees of polymerization are still present in the reaction system. These hinder the binding of substrate with MTSase, reducing maltodextrin utilization. Previous research has aimed to increase the trehalose conversion rate by enhancing the activity of *Sulfolobus acidocaldarius* maltooligosyltrehalose synthase via directed evolution. Separately expressed, or co-expressed, MTSase and MTHase from *Arthrobacter ramosus* S34 in *Escherichia coli* BL21 (DE3) was also attempted (Huang et al., 2024; Su et al., 2020). However, despite these attempts, the conversion rate remains relatively low, and many small maltooligosaccharides remain unused after the reaction is completed. Improved substrate utilization and higher trehalose yield has been obtained by integration of cyclomaltodextrin glucanotransferase (CGTase) into this system, which enhanced transglycosylation of *α*-1,4-glycosidic bonds utilizing low-molecular-weight maltose as a donor (Chen et al., 2020).

CGTase (EC 2.4.1.19) belongs to the α-amylase or glycosyl hydrolase family 13 and is categorized within the transferase (EC.2), transglycosylase (EC 2.4.) and hexosyltransferase subclasses (EC 2.4.) (Yang et al., 2022). CGTase is widely distributed among various *Bacillus* species such as *Paenibacillus macerans* (Chen et al., 2019), *B. circulans* (Huang et al., 2014), *B. megaterium* (Ji et al., 2022), *B. cereus* (Zhou et al., 2021), and *B. agaradhaerens* (Yang et al., 2022), where it catalyzes cyclization, disproportionation, coupling, and hydrolytic reactions (Pardhi et al., 2023). Specifically, its cyclization function transforms starch and its derivatives into cyclodextrins, which are crucial in various industrial applications (Szerman et al., 2007). Disproportionation and coupling reactions involve intermolecular transglycosylation, where the sugar group from the donor carbohydrate, containing hydroxyl or other functional groups, forms glycoconjugates. Disproportionation is dominant in these reactions, transferring cyclodextrins and maltooligosaccharides to substrates (Tao et al., 2020). This glycosylation increases hydrophilicity (Xiao et al., 2022), chemical stability (Song et al., 2021) or bioavailability (Lorthongpanich et al., 2022) of natural and industrial compounds. For example, glycosylation of hesperidin by CGTase, especially the more active recently obtained Y1305F/D2H mutant, yields *α*-glycosylhesperidin which has higher solubility (Chen et al., 2023). CGTase’s transglycosylation capability extends to elongating sugar chains in maltodextrin, further broadening its industrial applications.

The present study focused on enhancing the disproportionation activity of CGTase from *Bacillus* sp. G1 (BsCGT), particularly the synthesis of long-chain maltooligosaccharides through glycosylation with MOS as substrate. The objective was to ensure compatibility of CGTase catalytic properties with MTHase and MTSase reactions for trehalose production. To achieve this, the initial steps involved constructing a CGTase enzyme model via homology modeling. Subsequent docking of maltose substrates and simulation of amino acid mutations guided experimental validation aimed at refining CGTase’s disproportionation function. Concurrently, enhancing the enzyme’s thermal stability, particularly through mutations near the calcium ion binding site, led to the discovery of the N33K/S211G mutant. This variant demonstrated enhanced thermal stability and significantly increased trehalose production compared to the wild type. This study ascertained the substantial potential of engineered CGTase in enhancing the efficiency and cost-effectiveness of trehalose production, thereby promoting enzyme engineering and biocatalysis.

## 2 Materials and methods

### 2.1 Bacterial strains and plasmids

The CGTase gene derived from *Bacillus* sp. G1 (NCBI number: P31746) was obtained from the National Center for Biotechnology Information.^1^ *Escherichia coli* DH5α [F^−^, φ80dlacZ*Δ*M15, Δ(lacZYA-argF)U169, deoR, recA1, endA1, hsdR17(r_K_^−^, m_K_^+^), phoA, supE44, *λ*^−^, thi-1, gyrA96, relA1] was used for plasmid construction, and BL21 (DE3) {F^−^ ompT hsdS (r_B_^−^ m_B_^−^) dcm^+^ Tet^r^ galλ (DE3) endA Hte [argU proL Cam^r^] [argU ileY leuW Strep/Spec^r^]} was applied for gene expression. All plasmids used to express recombinant protein were derived from pET28a.

### 2.2 Homology modeling

**Figure 1:**
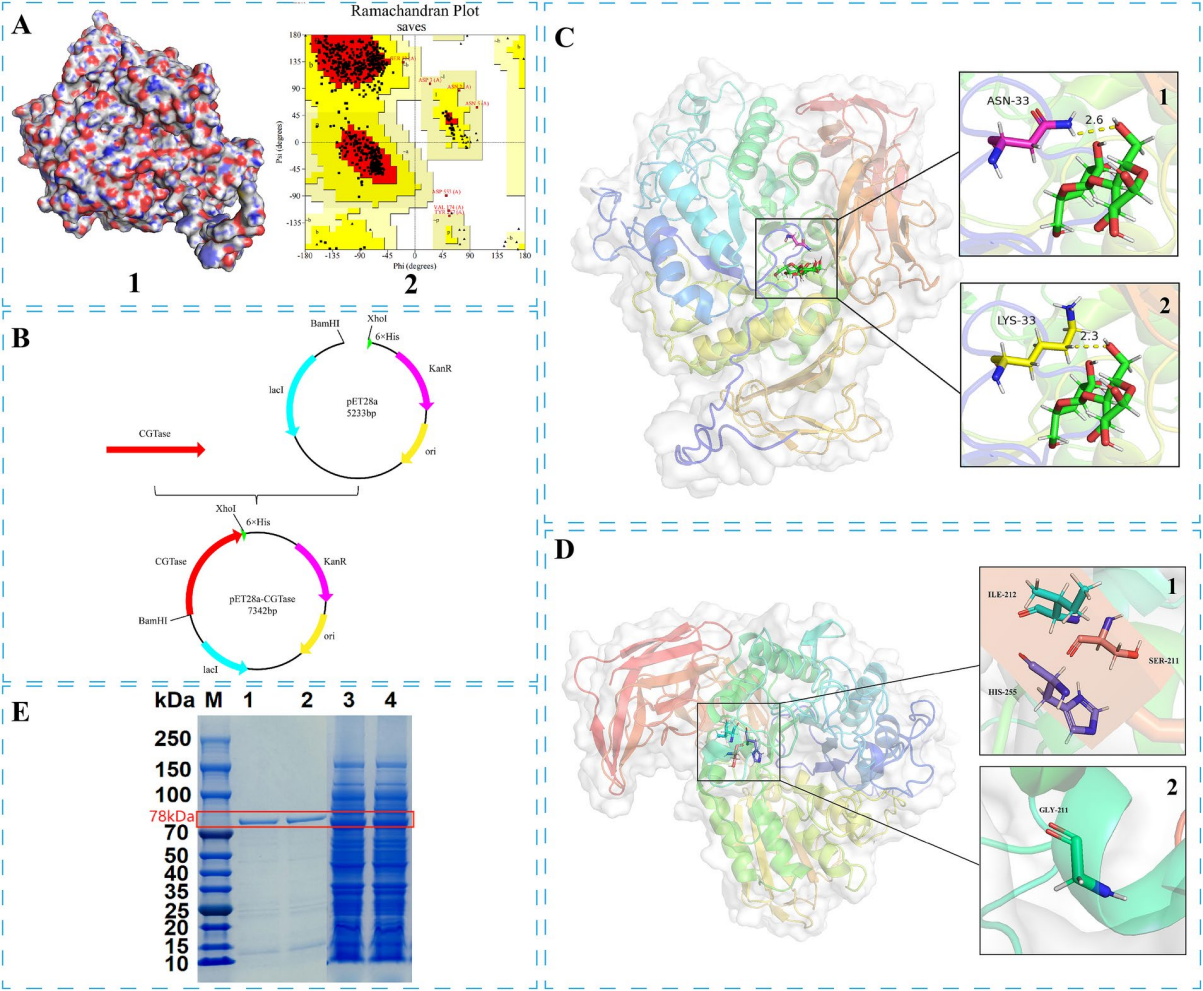
Modification process of disproportionation characteristics and thermal stability of *Bacillus* sp. G1 CGTase: (A1) 3-D structure of BsCGT; (A2) Ramachandran plot of BsCGT. (B) Construction of the recombinant plasmid. (C) Receptor-substrate docking model of the 3-D structure of wild-type BsCGT (C1) and mutant N33K (C2). (D) Display of mutation sites in the 3-D structure: (D1) Wild-type; (D2) S211G. (E) SDS-PAGE electrophoresis of the target protein and its purified form. Lane 1: N33K/S211G purified protein; Lane 2: N33K purified protein; Lane 3: N33K/S211G; Lane 4: N33K.

*Bacillus* sp. G1 CGTase (BsCGT) (Goh et al., 2012b) structure was obtained by comparison with the structure of *B. thermophilus* CGTase (PDB ID: 1CYG) (Song et al., 2023), which share 62.15% sequence similarity. Homology modeling using the SWISS-MODEL online service generated a three-dimensional structural model of BsCGT (Figure 1A1) which was employed in docking analyses. The structural integrity of the BsCGT model was assessed using the SAVES server.^2^

### 2.3 Construction of CGTase mutants

This study employed a one-step PCR method for targeted mutagenesis of plasmids. Specific primers for the CGTase gene from *Bacillus* sp. G1 facilitated targeted mutations, as detailed in Supplementary Table S1 (rows 1–27). The construction of thermostable mutant was initially carried out by importing the 3D structural model of CGTase was imported into PyMOL for structural analysis and image visualization. Discovery Studio was utilized to dock CGTase and calcium ions, identify amino acid residues near the binding site, conduct virtual mutations on selected residues, and calculate the energy change associated with these mutations near the binding site (Li et al., 2023). Mutations were performed on the plasmid using the one-step PCR method outlined. With the template containing the single mutation N33K, Primer sequences for these mutations are detailed in Supplementary Table S1 (rows 28–36). Following DpnI digestion to remove the template, the product was transformed into *E. coli* DH5α. The transformed cells were selected on Luria-Bertani (LB)-kanamycin (Kan) agar, cultured in LB-Kan liquid, and plasmid DNA was extracted using the TIANGEN kit for identification and sequencing. The mutant plasmids were then chemically transformed into *E. coli* BL21(DE3) (Figure 1B). This study analyzed enzyme expression at different fermentation temperatures.

### 2.4 Expression and purification of enzyme

The engineered mutant strains were stored at −80°C in glycerol and revived on LB-Kan plates overnight at 37°C. Selected colonies were grown in LB-Kan broth at 37°C, 200 rpm for 8–10 h. These starter cultures were then inoculated into TB-Kan broth at a 5% rate and incubated until OD600 reached 1.5–2.0. IPTG was added to induce expression, and the cultures were transferred to 25°C for 12 h of fermentation.

The mutant CGTase and wild-type CGTase were purified using Ni-NTA His Bind Resin (7SEA Biotech). The enzyme concentration was measured using the microplate method with a BCA kit (Boster Biological Technology).

### 2.5 Enzyme activity measurement

The disproportionation activity of CGTase was evaluated using the method described by van der Veen et al. (2000). The assay mixture included 12 mM 4-nitrophenyl-*α*-D-maltoheptaose-4,6-O-ethylidene (EPS) and 10 mM maltose, both obtained from Shanghai Yuanye Bio-Technology Co., Ltd. The procedure was initiated with a pre-incubation at 55°C for 10 min, followed by the addition of appropriately diluted CGTase for another 10 min at the same temperature. Afterwards, the reaction was incubated with 100 μL of α-glucosidase (10 U mL^−1^) (EC 3.2.1.20 from Shanghai Yuanye Bio-Technology Co., Ltd) at 37°C for 1 h. The reaction was terminated by adding 100 μL of 1 M sodium carbonate, and absorbance was measured at 401 nm. Enzyme activity, expressed in units per milliliter (U mL^−1^), is defined as the amount of enzyme that catalyzes the conversion of 1 μmol of EPS per minute (van der Veen et al., 2001).

### 2.6 Determination of enzymatic properties

#### 2.6.1 Impact of temperature and pH on enzyme activity

The effect of temperature on the activity of both wild-type and mutant CGTase enzymes was evaluated by conducting enzyme assays at different temperatures ranging from 35°C to 75°C for 10 min (Su et al., 2021). To determine the optimal pH for these enzymes, the standard 0.1 M phosphate buffer (pH 7.2) used in CGTase assays was replaced with various other buffers: 0.1 M sodium acetate (pH 4.0–5.0), 0.1 M potassium phosphate (pH 6.0–8.0), and 0.1 M glycine-NaOH (pH 9.0–10.0) (Liu et al., 2022).

The thermal stability of the enzyme was tested by incubating 0.1 mL of the purified enzyme with a substrate solution at pH 6.0 across a range of temperatures (35–90°C) for 1 h. Additionally, CGTase was evaluated after a 1 h incubation at 55°C in buffer solutions with pH values ranging from 4.0 to 9.0, without substrates present. The residual activities were measured according to the enzyme activity assay described in Section 2.6, with the initial activity under optimal conditions set as 100%.

#### 2.6.2 Differential scanning calorimetry

Thermal denaturation of CGTase was analyzed using a DSC 240 F1 (NETZSCH., Germany). DSC analysis was conducted over a temperature range of 30–90°C, with a temperature increase rate of 1°C per minute. The sample concentration was maintained at 1 mg mL^−1^. The instrument was equilibrated for 10 min prior to starting the scan.

### 2.7 Determination of kinetic parameters

Maltose solutions at concentrations of 0.25, 0.5, 0.75, 1, 1.5, 2, 3, 5, 10, 20, 50, and 100 mM were prepared and preheated. The enzyme activity at these varying substrate concentrations was assessed under optimal reaction conditions: pH 6.0 and a temperature of 55°C, using the method described in Section 2.5. The resulting data were analyzed using GraphPad Prism software for curve fitting, from which kinetic constants were derived.

### 2.8 CGTase in trehalose production from maltodextrin

For the synthesis of trehalose, the purified fusion enzymes, Sase-CcDoc and CtDoc-Hase, along with the scaffold protein ScafCCR, were initially assembled into a bi-enzyme complex system. Specifically, they were mixed at an equimolar ratio and underwent a reaction in a constant-temperature water bath at 37°C for 2 h (Wang et al., 2023). Subsequently, 200 g L^−1^ maltodextrin (with a glucose equivalent of 9) was employed as the substrate. The assembled double enzyme was added, in conjunction with 5 U g^−1^ Pullulanase, and the reaction lasted for 48 h at 60°C, with regular sampling. Moreover, CGTAses were introduced and samples were collected at regular intervals (Chen et al., 2020). To inactivate the enzymes, the sample was boiled for 10 min following the reaction, followed by the addition of amylase, and then shaken in a water bath at 60°C for 6 h. Eventually, the mixture was boiled for another 10 min to ensure the complete deactivation of the enzymes.

### 2.9 Concentration detection of trehalose

After the reaction was completed, the resulting solution was centrifuged at 20,000 × g for 10 min to separate the supernatant, which was then appropriately diluted. The trehalose content in the conversion solution was quantified using high-performance liquid chromatography (HPLC). This was performed on a Shimadzu HPLC system (LC-10ATV) equipped with an Inertsil NH₂ chromatography column (4.6 mm × 250 mm, 5 μm, GL Sciences (Shanghai) Co., Ltd). The analysis conditions included a mobile phase of acetonitrile to water in a 4:1 ratio, a flow rate of 1.0 mL/min, and a column temperature of 40°C (Trakarnpaiboon and Champreda, 2022).

### 2.10 Statistical analysis of data

Each experiment included three replicates. Results are presented as the average value ± standard deviation (SD). Statistical evaluations were performed using one-way analysis of variance (ANOVA). The Origin 9.4 software platform (OriginLab Corporation, United States) was utilized for statistical analysis. Post-hoc comparisons of means were conducted using Tukey’s Honest Significant Difference (HSD) test to identify statistically significant differences between groups.

## 3 Results

### 3.1 Structural bioinformatics of CGTase

Evaluation of the Ramachandran plot of the BsCGT model (Figure 1A2) obtained from homologous modeling, indicated that the model was credible, since 86.6% of residues fell within the most favored region, 99.7% in allowed regions and only 0.3% in disallowed regions. To identify mutations that enhance substrate affinity, this enzyme model was docked with maltose, and the amino acids near the substrate binding site were identified according to the docking results. An alanine scan is then performed to determine which amino acids may change affinity when mutated. Virtual saturation mutations of these amino acids were subsequently performed, and the results showed that mutations at Asn33, Tyr119, Tyr122, Leu216, His255, Glu258, Pro394, and Glu566 may be conducive to improving affinity (Table 1).

**Table 1:** Virtual amino acid mutation screening.

Mutant amino acids	Secondary structure	Intermolecular force	Mutation energy
Asn33 > Arg	Loop	Carbonl-hydrogen bond	−1.74
Asn33 > Lys	Loop	Carbonl-hydrogen bond	−1.3
Tyr119 > Arg	Loop	Carbonl-hydrogen bond	−0.72
Tyr122 > Glu	Loop	Carbonl-hydrogen bond	−0.91
Leu216 > Gln	Loop	Carbonl-hydrogen bond	−0.52
His255 > Tyr	α-helix	Carbonl-hydrogen bond	−0.56
Glu258 > Tyr	α-helix	Carbonl-hydrogen bond	−1.03
Glu258 > Gln	α-helix	Carbonl-hydrogen bond	−0.96
Pro394 > Arg	Loop	Carbonl-hydrogen bond	−0.88
Pro394 > Gln	Loop	Carbonl-hydrogen bond	−0.80
Glu566 > His	β-pleated sheet	Carbonl-hydrogen bond	−0.77

### 3.2 Expression and enzymatic properties of mutase N33K

Enzyme expression was tested under different fermentation temperatures, revealing that cultivation at 25°C yielded optimal enzyme activity (Supplementary Figure S1). Thus enzymatic activity was evaluated for several single-point mutants, identifying mutations with advantageous characteristics. These mutations underwent further mutagenesis and activity results are shown in Supplementary Table S2. Among these, mutant N33K showed an activity (20.33 U mL^−1^) that was significantly higher than the one of the wild type (12.60 U mL^−1^) and identified as the optimal mutant.

The molecular mass of the recombinant mutase N33K, produced through fermentation, was determined using SDS-PAGE after nickel column purification (Figure 1E). Subsequent characterization of the N33K mutant included assessing its optimal temperature, thermal stability, optimal pH, and pH stability. These investigations revealed that the amino acid changes did not significantly alter these enzymatic performance characteristics (Supplementary Figure S2).

Table 2 presents the kinetic constants using maltose as the substrate for both wild-type BsCGT enzyme and its N33K mutant variant. The wild-type BsCGT showed a catalytic constant (*k*_cat_) of 22.81 s^−1^ and a Michaelis–Menten constant (*K*_M_) of 16.63 mM, indicating efficient catalytic activity and substrate binding affinity. The N33K mutant exhibited a reduced *K*_M_ value (14.47 mM), indicating a stronger binding affinity for maltose, as it requires less substrate concentration to achieve half the maximum reaction velocity. The mutant also showed a higher *k*_cat_ (36.17 s^−1^), indicating an increased turnover number and enhanced ability to rapidly convert substrate to product.

**Table 2:** Enzymatic kinetic parameters of wild-type BsCGT and mutant^*^.

Enzyme	*K*_M_ (mM)	*k*_cat_ (s^−1^)	*k*_cat_/*K*_M_ (s^−1^·mM^−1^)
BsCGT	16.63 ± 0.24^a^	22.81 ± 0.05^b^	1.37 ± 0.02^b^
N33K	14.47 ± 0.19^b^	36.17 ± 0.08^a^	2.50 ± 0.03^a^
N33K/S211G	14.20 ± 0.21^b^	36.34 ± 0.11^a^	2.56 ± 0.14^a^

^a–b^Values with different letters within the same column are significantly different (*p* < 0.05). ^*^Each value represents the mean ± standard deviation of three independent measurements. K_M_, Michaelis constant; k_cat_, catalytic constant or turnover number, k_cat_/K_M_, specificity constant (ratio k_cat_/K_M_).

Molecular dynamics simulations were performed to compare the substrate interactions of the CGTase N33K mutant and its wild-type counterpart. The simulations revealed that the N33K mutation, replacing asparagine with lysine at position 33, introduces an additional positive charge in the enzyme’s active site. This charge modification enhances electrostatic interactions with the negatively charged regions of maltose, leading to the formation of more stable hydrogen bonds. Specifically, in the N33K mutant (Figure 1C2), the distance between lysine-33 and maltose was reduced to 2.3 Å, whereas in the wild-type (Figure 1C1), this distance was 2.6 Å. This closer interaction is likely facilitated by lysine’s side chain, which can form hydrogen bonds with the hydroxyl groups of maltose. These interactions are expected to improve substrate binding and stabilize the transition state, potentially lowering the activation energy required for the catalytic reaction.

### 3.3 Bioinformatics analysis of CGTase thermal stability

In industrial processes where high operating temperatures were required, the development of heat-resistant CGTase was of paramount importance. The addition of CaCl_2_ was an effective method to enhance the enzyme’s thermal tolerance, which improved CGTase activity and stability. However, the presence of CaCl_2_ could complicate downstream processing, posing challenges for industrial applications. CGTase’s stability was strongly influenced by intermolecular interactions, particularly those involving calcium ions. These interactions played a crucial role in maintaining the enzyme’s structural integrity and functional stability under elevated temperatures. However, the specific regions or residues with CGTase that contributed to its thermal stability were still under investigation and required further elucidation. Understanding the molecular mechanisms underlying CGTase’s thermal stability, particularly in the presence of CaCl_2_, was essential for developing strategies to engineer enzymes with enhanced thermal tolerance. This knowledge could guide the design of CGTase variants that maintained robust performance under harsh industrial conditions while minimizing the need for additives like CaCl_2_ during downstream processing. Calcium ions play a crucial role in stabilizing CGTase through coordination bonds with oxygen-containing amino acid residues such as Asp., Glu, Ser and Thr.

Sequential alignment of CGTase from various sources was conducted to identify critical residues that enhance thermal stability. This approach, depicted in Supplementary Figure S3, emphasized calcium-interaction sites (highlighted in a green box) and specific regions (marked in a red box) previously recognized as crucial for temperature adaptability. The modifications, within these identified areas could significantly prolong the enzyme’s half-life (Leemhuis et al., 2004).

Insights led to the selection of Ser211, Ile212, and His255 for mutagenesis due to their proximity to the calcium-binding site. The hypothesis was that mutating these residues could alter the enzyme’s interaction with calcium ions, potentially affecting its thermal stability. Utilizing directed evolution, five promising mutations were identified: S211G, I212R, H255W, H255F, and H255Y, as shown in Figure 1D1. These changes were anticipated to impact the enzyme’s thermal stability by modulating its calcium-binding affinity.

### 3.4 Expression and enzymatic properties of the thermostable mutants

The integrity of purified CGTase was confirmed via SDS-PAGE (Fiure 1E). The enzymatic activities of the thermostable double mutants containing mutation N33K (Table 3) show that disproportionation activity was reduced in mutants N33K/H255W, N33K/H255F and N33K/H255Y, where His255 was substituted. This suggests that His255 plays a critical role in either substrate binding or in catalysis. In contrast, the activities of the N33K/S211G and N33K/I212R mutants remained largely unchanged.

**Table 3:** Enzyme activity of thermostable mutants using N33K mutant as a template^*^.

Enzyme	Enzyme activity (U mL^−1^)
BsCGT	12.60 ± 0.14^d^
N33K	20.33 ± 0.22^b^
N33K/S211G	24.72 ± 0.11^a^
N33K/I212R	24.48 ± 0.10^a^
N33K/H255W	10.04 ± 0.09^e^
N33K/H255F	13.50 ± 0.12^c^
N33K/H255Y	10.22 ± 0.10^e^

^a–e^Values with different letters within the same column are significantly different (*p* < 0.05). ^*^Each value represents the mean ± standard deviation of three independent.

The optimal pH and pH stability for N33K, N33K/S211G, and N33K/I212R mutants was similar to that of the wild type enzyme (Supplementary Figure S2).

**Figure 2:**
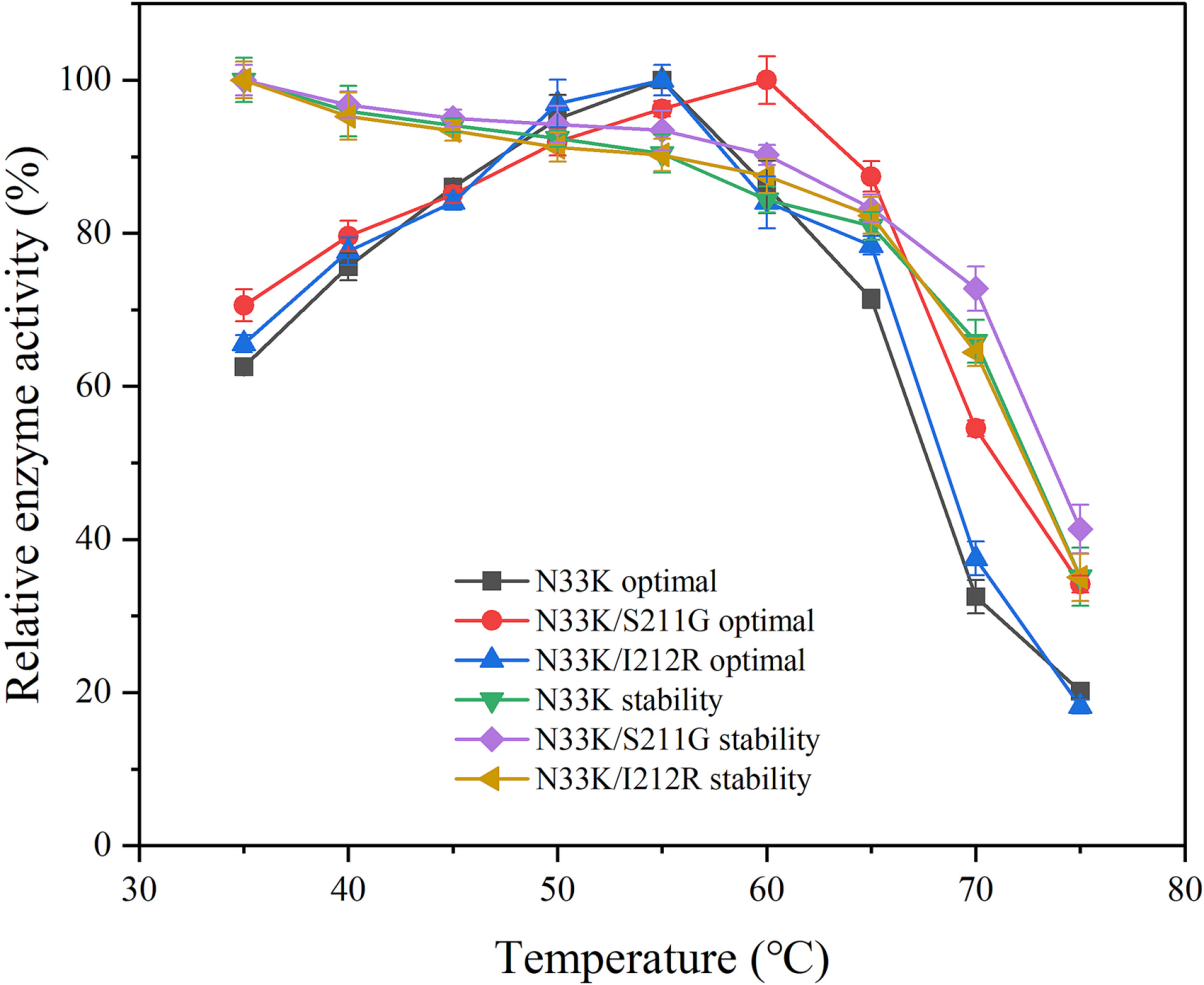
Optimal temperature and thermal stability of mutant CGTases.

Enhancing the optimal temperature and temperature stability of enzymes is crucial for their application, noting that enzymatic activity peaks at this optimum temperature. Setting the activity at the optimal temperature for mutants and wild-type BsCGT to 100%, the optimal temperature for N33K/S211G was 60°C, whereas for N33K, N33K/I212R, and wild-type BsCGT, it remained at 55°C (Figure 2). The introduction of the N33K/S211G mutation effectively raised the enzyme’s optimum temperature to 60°C. The same figure compares the thermal stability of these mutants which were subjected to various temperatures for 1 h. Whereas mutant N33K/I212R and N33K showed similar thermal stability profiles, N33K/S211G showed higher stability. Specifically, the half-life of N33K/S211G at temperatures of 50, 55, 60, and 65°C was 2.4 h, 2.0 h, 55.4 min, and 38.4 min, respectively. For N33K/I212R, the half-lives at these temperatures were 1.9 h, 1.4 h, 25.2 min, and 21.0 min (Supplementary Figure S4), respectively. Notably, at the optimal temperature of 60°C, the half-life of N33K/S211G was 2.3-fold longer than that of N33K (24.0 min). This highlights the significant contribution of the Ser211 mutation (Figure 1D) in N33K/S211G to structural stabilization and improved thermal stability of CGTase. Mutations at this site critically influence the enzyme’s overall activity and temperature adaptability to high temperatures, offering insights into enhancing its performance for industrial applications.

The thermal stability of the mutants was also assessed using Differential Scanning Calorimetry (DSC), which measures heat change associated with conformational transitions in a temperature ramp. The T_m_ value, derived from the DSC curve, represents the temperature at which the protein transitions from a folded state to an unfolded state, which is a critical indicator of thermal denaturation. For the wild-type BsCGT, T_m_ was 65.2°C, whereas it was higher for mutant N33K/S211G (67.8°C).

### 3.5 CGTase-assisted trehalose preparation

**Figure 3:**
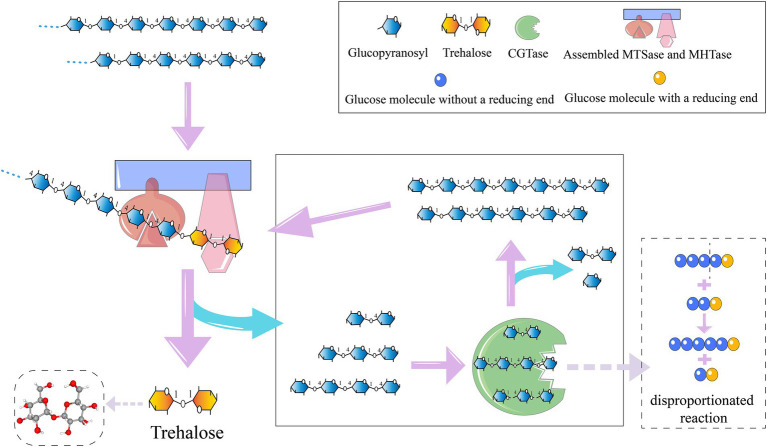
Trehalose preparation process.

**Figure 4:**
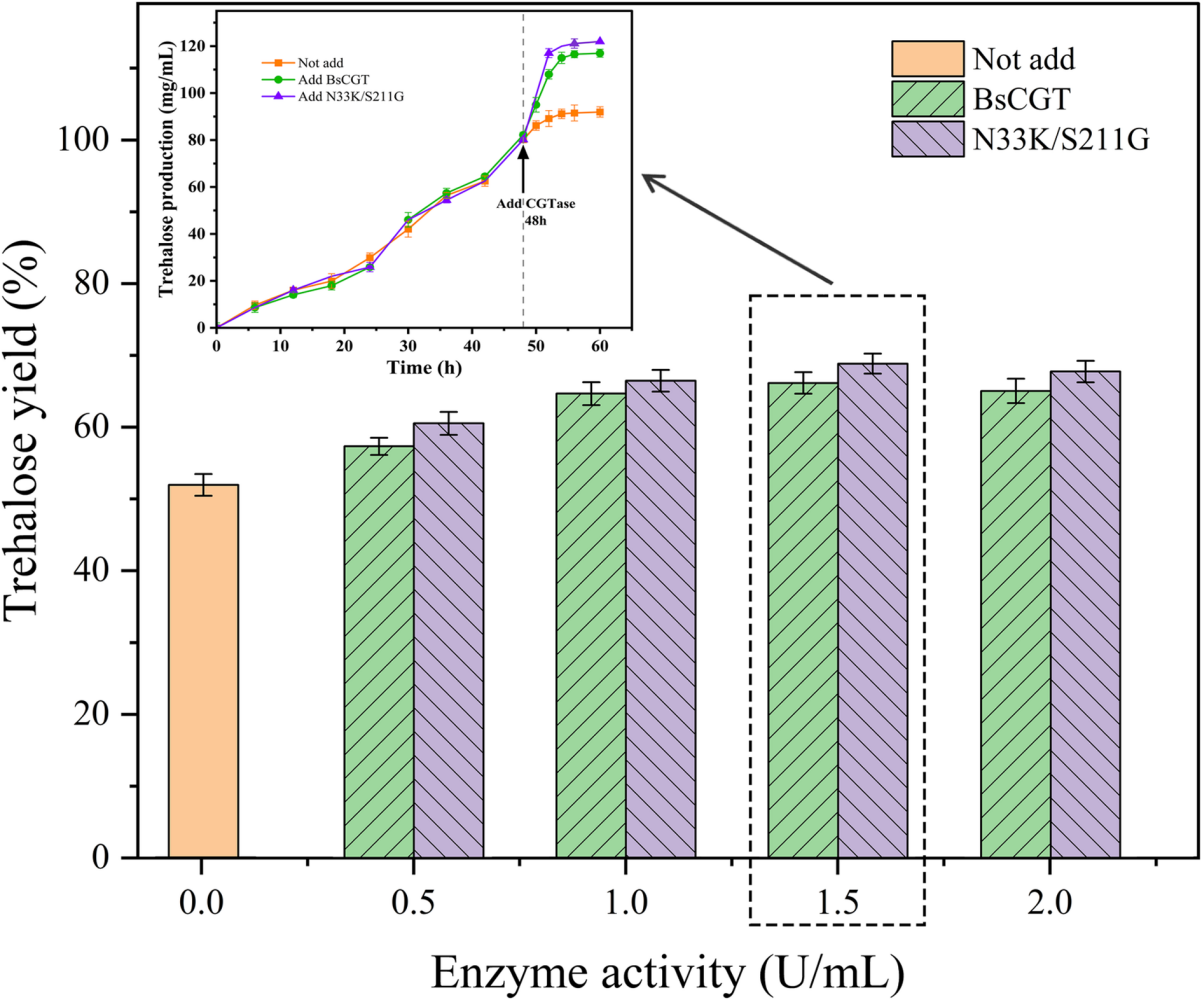
Trehalose conversion rate at different enzyme doses and trehalose yield at optimal enzyme dose.

Trehalose synthesis was achieved using laboratory-prepared MTSase and MTHase enzymes with the incorporation of either wild-type BsCGT or mutant enzyme N33K/S211G toward the end of the reaction schematically shown in Figure 3. Addition of CGTase increased trehalose production by 20% (Figure 4), and trehalose yield was higher using the N33K/S211G mutant compared to BsCGT. For example, at an enzyme concentration of 1.5 U mL^−1^, the mutant achieved a conversion of 69%, higher than 66% achieved by wild-type BsCGT. Additionally, the analysis of the reaction product of trehalose preparation by BsCGT indicated that the maltose content in the N33K/S211G mutant (5.01 g L^−1^) was about 33.6% lower than that in the wild-type BsCGT (7.54 g L^−1^). This may be attributed to the mutant’s enhanced affinity for maltose, allowing it to convert maltose to maltodextrin more efficiently, thereby improving substrate utilization. Therefore, this is also consistent with the difference in trehalose yield in the reaction product composition analysis, indicating that the mutant can increase trehalose yield compared with the wild enzyme (Table 4).

**Table 4:** Product composition during trehalose preparation^*^.

Enzyme	Composition of each component (g L^−1^)		
	Trehalose	Maltose	Glucose
BsCGT	112.58 ± 1.43^b^	7.54 ± 0.21^a^	27.43 ± 0.44^c^
N33K	116.82 ± 1.07^a^	5.77 ± 0.27^b^	29.16 ± 0.57^b^
N33K/S211G	118.21 ± 0.99^a^	5.01 ± 0.22^c^	31.73 ± 0.60^a^

^a–c^Values with different letters within the same column are significantly different (*p* < 0.05). ^*^Each value represents the mean ± standard deviation of three independent measurements.

Overall, integration of homology modeling, docking analysis and virtual mutagenesis is a robust approach to understand and improve the structural and functional characteristics of BsCGT, and to advance enzyme engineering in industrial biotechnological applications.

## 4 Discussion

When trehalose is prepared from maltodextrin using the double-enzyme approach, both the substrate and the product of the reaction process contain a considerable amount of maltose oligosaccharide, which significantly contributes to the high production cost. Although some studies have been conducted to reduce this waste by adding glycosyltransferase, there is still insufficient clarification of the mechanism and effectiveness of reusing oligomertodextrin. In this study, firstly, to better understand the relationship between the structure and function of the CGTase (Goh et al., 2012b) of *Bacillus* sp. G1 (BsCGT), the CGTase (PDB ID: 1CYG) derived from *Bacillus thermophilus* (Song et al., 2023) was used as a template for structural comparison. The three-dimensional structural model constructed through homologous modeling was imported into Discovery Studio and docked with the substrate maltose molecule. Virtual amino acid mutations were performed in the substrate catalytic region based on the docking results.

It has been reported that CGTase can be secreted in *Escherichia coli* and secreted into the extracellular culture medium due to the existence of a series of signaling peptides on the gene encoding *Bacillus subtilis* (Ong et al., 2008). BsCGT was successfully expressed in a secretory form in *Escherichia coli*, followed by site-specific mutation. The resultant mutant, upon successful expression, was compared with the wild-type BsCGT, and the N33K mutant exhibited a remarkable improvement in enzymatic kinetics. The significant increase in *k*_cat_ indicates an optimized catalytic mechanism, which might be attributed to the reduction of the energy barrier of the transition state or the stabilization of the enzyme-substrate complex, thereby accelerating product formation. Molecular dynamics simulations provide valuable insights into the structural and energetic factors that underlie the enhanced catalytic efficiency of the N33K mutant enzyme. The augmented capacity for hydrogen bonding, facilitated by lysine, not only enhances the initial substrate binding but also stabilizes the transition state during catalysis. Furthermore, the N33K mutation induces conformational alterations, particularly in the loop region surrounding the active site. These changes probably optimize the enzyme’s structure to accommodate the substrate more effectively. This precise alignment might restrict the substrate’s mobility, minimizing dissociation events and thereby enhancing the overall catalytic efficiency of the enzyme.

The study of enzymatic properties holds crucial industrial value for the efficacy of the application of the relevant properties of industrial enzyme preparations (Zheng et al., 2019). The thermal stability of enzymes is indispensable for enzymatic reactions and constitutes one of the significant properties of enzymes (Goh et al., 2012a). In reality, in the reaction system, numerous factors influence the thermal stability of enzymes. There were diverse reasons for the alteration in enzyme thermal stability, and the most significant reason was the change in its spatial structure. Enzymes with high thermal stability contributed to enhancing the catalytic efficiency in the reaction system, which reduced the production cost and simplified the process to a certain extent and had an influence on the stability of product quality. The study reported that CGTase from *Thermobacterium thermosulfurigenes* EM1 exhibited high thermal stability, with a half-life of up to 15 min at 90°C (Goh et al., 2012a). CGTase from *Bacillus circulans* 251 was not a heat-resistant enzyme, having a half-life of 9.7 min at 60°C (Leemhuis et al., 2004), while CGTase from *Brevibacillus brevis* CD162 had a half-life of 30 min at 55°C (Ong et al., 2008). In this study, we investigated the temperature stability of the optimal mutation N33K and the wild-type BsCGT. After repeated experiments, it was discovered that the obtained mutant N33K exhibited no significant heat resistance, and thus its thermal stability was enhanced.

The obtained mutant N33K/S211G, and the determination results of enzymatic properties suggest that the amino acid substitution has increased the stability of the mutant enzyme, as it begins to denature at a higher temperature compared to the wild-type. The enhanced stability observed in the mutant is likely attributed to the impact of the amino acid substitution on internal protein interactions, potentially strengthening hydrogen bonds, hydrophobic interactions, or ionic bonds within the protein structure.

When the mutant N33K/S211G was added to the trehalose preparation process, the maximum conversion rate reached 68.7%. In the analysis of the reaction product, it was discovered that the content of maltose was significantly decreased, suggesting that the affinity of the mutant N33K to maltose was enhanced. As we had predicted earlier, this enables maltose and oligomer todextrin to be reused for the production of more long-chain maltodextrin. Long-chain maltodextrin is utilized by the double enzyme system to generate trehalose, reducing substrate waste and production costs, and enhancing trehalose conversion and yield.

## 5 Conclusion

This study acquired the recombinant mutant N33K/S211G, which showed a 2.1-fold enhancement in transglycosylation activity over the wild type. This mutant enhanced thermal stability and adaptability, resulting in an increased optimal temperature to 60°C and a prolonged half-life at this temperature. It was more effective in the dual-enzyme trehalose production system from maltodextrin, achieving a conversion of 68.7%. The study emphasizes the industrial potential of recombinant BsCGT mutants for increasing trehalose yield and offers novel insights for CGTase engineering and trehalose industrialization.

## Data Availability

The original contributions presented in the study are included in the article/Supplementary material, further inquiries can be directed to the corresponding authors.

## Footnotes

^1^ http://www.ncbi.nlm.nih.gov/

^2^ https://saves.mbi.ucla.edu/

## Supplementary material

The Supplementary material for this article can be found online at:

https://www.frontiersin.org/articles/10.3389/fmicb.2024.1500232/full#supplementary-material
