# Supplementary material for: Enhancing trehalose production via Bacillus species G1 cyclodextrin glucanotransferase mutants: modifying disproportionation characteristics and thermal stability
Source: Front Microbiol. 2024 Nov 19;15:1500232. doi: 10.3389/fmicb.2024.1500232 (PMC11611815; doi:10.3389/fmicb.2024.1500232)
Supplement: Supplementary file 1 [file Table_1.docx]

Supplementary Material

**Table S1 Primers used for site-directed mutagenesis.**

| Sequence | Primer | Sequence from 5’ to 3’(Forward)^*^ |
| --- | --- | --- |
| 1 | N33K-F | GTTACAAAGAAAGTCAATTATTCTAAGGATGTGATTTACC |
| 2 | N33K-R | TGACTTTCTTTGTAACATCTGCTTCAGCTACTG |
| 3 | N33R-F | GTTACAAGGAAAGTCAATTATTCTAAGGATGTGATTTACC |
| 4 | N33R-R | GACTTTCCTTGTAACATCTGCTTCAGCTACTG |
| 5 | Y119R-F | TCACGTCATGGCTATTGGGCAAGAGA |
| 6 | Y119R-R | GCCATGACGTGATGTATAGCCGCTCGG |
| 7 | Y122E-F | GCGAATGGGCAAGAGATTATAAAAAAACAAATCCGT |
| 8 | Y122E-R | TTGCCCATTCGCCATGATATGATGTATAGCCGC |
| 9 | L216Q-F | AGAAATCAGTATGATTTGGCAGACTACGAT |
| 10 | L216Q-R | GCCAAATCATACTGATTTCTATAAATTGAATCTTCATATGA |
| 11 | H255Y-F | GTTAAATACATGTCAGAAGGCTGGCAAA |
| 12 | H255Y-R | CTGACATGTATTTAACTGCATCAACTCTAATGCCAT |
| 13 | E258Y-F | GTCATACGGCTGGCAAACATCACTG |
| 14 | E258Y-R | CAGCCGTATGACATATGTTTAACTGCATCAACTC |
| 15 | E258Q-F | GTCACAAGGCTGGCAAACATCACTG |
| 16 | E258Q-R | CAGCCTTGTGACATATGTTTAACTGCATCAACTCT |
| 17 | P394R-F | GATCGGGAAAATAGAAAACCGCTGAAAACA |
| 18 | P394R-R | CTATTTTCCCGATCATTGCCGCCCGTAACA |
| 19 | P394Q-F | CAATGATCAGGAAAATAGAAAACCGCTGAAAACA |
| 20 | P394Q-R | ATTTTCCTGATCATTGCCGCCCGTAAC |
| 21 | E566H-F | CAAATACACACATTTCAGTTAAAGTTCCGAATGTTGCGG |
| 22 | E566H-R | GAAATGTGTGTATTTGACCATGAAATAATTTCTGATGATG |
| 23 | CGTase-F | AAGAAGGAGATATACCATGAATGATCTGAATGATTTTC |
| 24 | CGTase-R | GTGGTGGTGGTGCTCGAGCCAATTAATCATAACTG |
| 25 | pET28a-F | GAAGGAGATATACCATGGGCAGCAG |
| 26 | pET28a-R | TTTAGAGGCCCCAAGGGGTTATGCT |
| 27 | S211G-F | GAAGATGGAATTTATAGAAATCTGTATGATTTGGCAGACT |
| 28 | S211G-R | TATAAATTCCATCTTCATATGATGAAAAATCTGTGCCGCC |
| 29 | I212R-F | GATTCAAGGTATAGAAATCTGTATGATTTGGCAGACTA |
| 30 | I212R-R | TCTATACCTTGAATCTTCATATGATGAAAAATCTGTGCCG |
| 31 | H255W-F | GTTAAATGGATGTCAGAAGGCTGGC |
| 32 | H255W-R | CTGACATCCATTTAACTGCATCAACTCTAATGCC |
| 33 | H255F-F | GTTAAATTTATGTCAGAAGGCTGGCAAACATC |
| 34 | H255F-R | CTGACATAAATTTAACTGCATCAACTCTAATGCCATC |
| 35 | H255Y-F | TAAATACATGTCAGAAGGCTGGCAAACATC |
| 36 | H255Y-R | CTGACATGTATTTAACTGCATCAACTCTAATGCC |

^*^ Underlined sequences indicate introduction of mutation.

**Table S2 Enzymatic activity of CGTase disproportionated single point and multi-site mutants*.**

| Sequence | Enzyme | Enzyme activity  (U mL^-1^) |
| --- | --- | --- |
| 1 | BsCGT | 12.60±0.14^f^ |
| 2 | N33K | 20.33±0.22^a^ |
| 3 | N33R | 17.25±0.30^e^ |
| 4 | Y119R | 12.29±0.22^f^ |
| 5 | Y122E | 11.92±0.43^fg^ |
| 6 | L216Q | 10.60±0.23^h^ |
| 7 | H255Y | 9.61±0.15^i^ |
| 8 | E258Y | 19.46±0.16^bc^ |
| 9 | E258Q | 19.49±0.20^bc^ |
| 10 | P394R | 19.72±0.15^abc^ |
| 11 | P394Q | 10.63±0.16^h^ |
| 12 | E566H | 11.49±0.23^g^ |
| 13 | N33K/E258Q | 20.11±0.18^ab^ |
| 14 | N33K/P394R | 18.23±0.27^d^ |
| 15 | E258Q/P394Q | 19.11±0.30^c^ |
| 16 | N33K/E258Q/P394Q | 9.23±0.13^i^ |

^a-i^ Values with different letters within the same column are significantly different (p < 0.05).

*Each value represents the mean ± standard deviation of three independent measurements.





**Figure S1 Effect of induction temperature on enzymatic activity.**


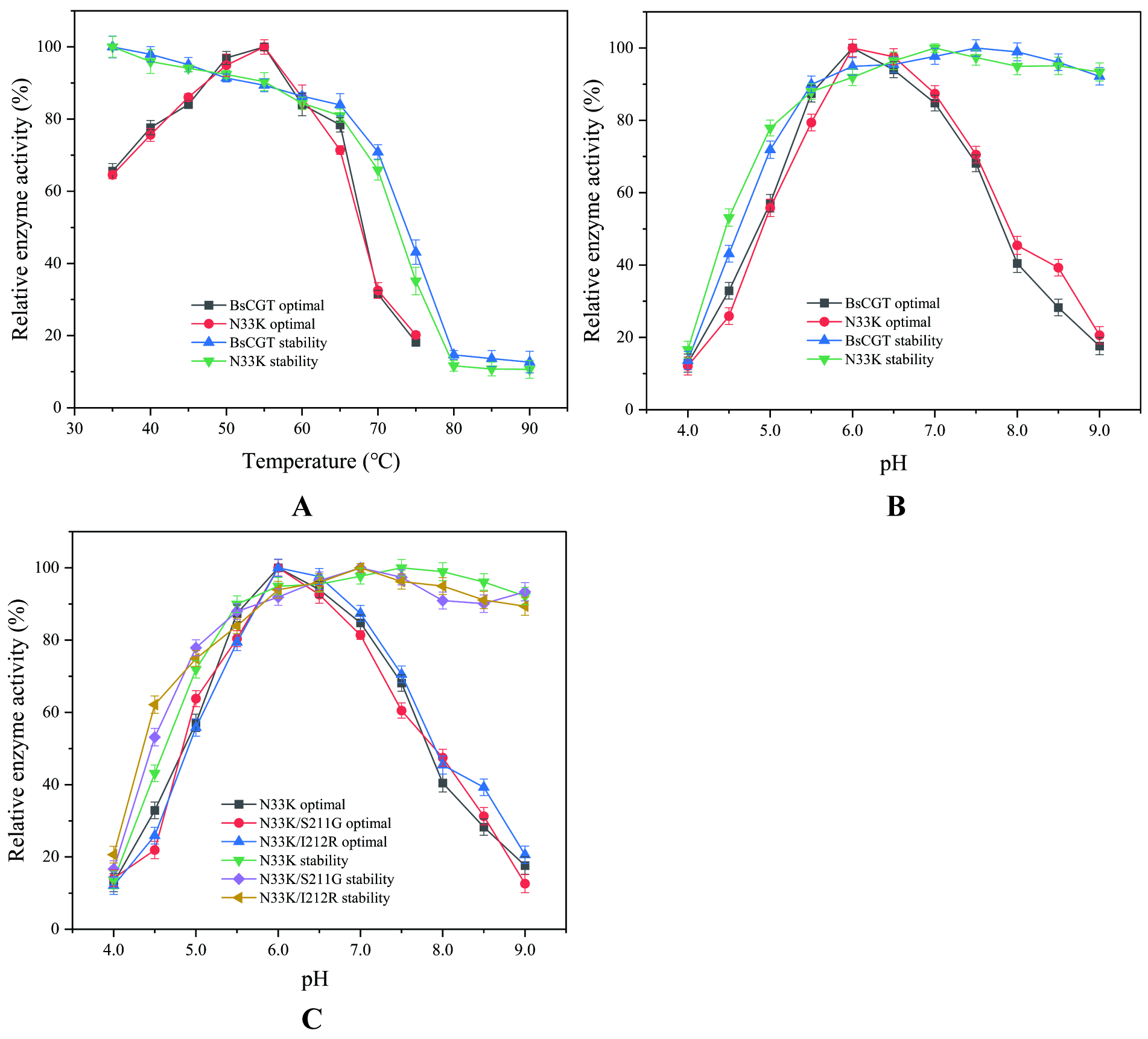


**Figure S2 Properties of BsCGTase and mutant.** A, Optimal temperature of BsCGT and N33K; B, Optimal pH of BsCGT and N33K; C, Optimal pH of N33K, N33K/S211G and N33K/I212R.

**Figure S3 CGTase sequence alignment from different sources.** *B. stearophilus* (1CYG); *Thermoanaerobacterium thermosulfurigenes* (1CIU); *B. ccirculans* 251 (1CXI). Revealed calcium-interacting sites (indicated by the green box) and regions (red box).

**Figure S4 Half-lives of N33K/S211G and N33K/I212R at temperatures of 50, 55, 60 and 65 ℃.** A, 50℃; B, 55℃; 60℃; D, 65℃.
